# The Beneficial Effects of Glucagon-Like Peptide-1 Agonists on Blood Pressure: A Comprehensive Review

**DOI:** 10.31083/RCM45204

**Published:** 2025-12-18

**Authors:** Dhir Gala, Fady Botros, Amgad N. Makaryus

**Affiliations:** ^1^Department of Medicine, Rutgers New Jersey Medical School, Newark, NJ 07103, USA; ^2^Department of Cardiology, Cardiovascular Institute, Northwell Health, New Hyde Park, NY 11040, USA; ^3^Department of Cardiology, Donald and Barbara Zucker School of Medicine at Hofstra/Northwell, Hempstead, NY 11549, USA

**Keywords:** hypertension, glucagon-like peptide-1 receptor agonists, diabetes, cardiovascular disease, weight loss, metabolic syndrome

## Abstract

Hypertension is a prominent cardiovascular risk factor, especially among patients with diabetes and obesity. Glucagon-like peptide-1 receptor agonists (GLP-1 RAs) are a class of drugs originally developed to improve glycemic control in patients with diabetes; however, these agonists have subsequently demonstrated additional cardioprotective effects, including modest reductions in blood pressure (BP). This literature review examines the various mechanisms through which GLP-1 RAs reduce BP, including weight loss, improved endothelial function, and renal sodium management. While GLP-1 RAs are less potent in BP reduction compared to conventional antihypertensive agents, the broader metabolic benefits of these agonists make this class of drug a valuable adjunct in managing hypertension, particularly in patients with metabolic syndrome. Nonetheless, further studies are needed to explore the long-term effects of BP and optimize patient selection for maximal cardiovascular benefit.

## 1. Introduction

Hypertension is a well-established major risk factor for cardiovascular diseases (CVD), often coexisting with metabolic disorders like type 2 diabetes mellitus (T2DM), compounding their cardiovascular risk [1]. Glucagon-like peptide-1 receptor agonists (GLP-1 RAs) were originally developed as antidiabetic agents to improve glycemic control. Beyond glucose lowering, GLP-1 RAs have been shown to confer broad cardioprotective benefits including reductions in body weight, blood pressure, postprandial lipids, and inflammation [2]. These agents mimic the incretin hormone GLP-1, enhancing insulin secretion, suppressing glucagon release, and delaying gastric emptying, making them effective in addressing hyperglycemia while exhibiting favorable effects on other metabolic parameters [3, 4]. Their impact on blood pressure regulation represents a critical area of exploration, given the interconnected relational nature of diabetes, obesity, and hypertension.

In diabetes management, GLP-1 RAs have been transformative due to their ability to achieve robust reductions in glycated hemoglobin (HbA1c) levels without significantly increasing the risk of hypoglycemia [5, 6]. These agents facilitate glucose-dependent insulin secretion, providing more physiological glycemic control compared to traditional therapies. Beyond glucose lowering, GLP-1 RAs induce satiety and promote weight loss, addressing the obesity-diabetes nexus that exacerbates insulin resistance and complicates disease management [7, 8]. Importantly, GLP-1 RAs also demonstrate reno-protective properties. Clinical trials and mechanistic studies suggest they reduce intraglomerular pressure through natriuretic effects, lower albuminuria, and attenuate the progression of diabetic kidney disease by reducing renal oxidative stress and inflammation [9, 10]. The broad spectrum of benefits offered by GLP-1 RAs underscores their value as foundational therapies in the management of diabetes and its complications.

The antihypertensive effects of GLP-1 RAs are thought to be mediated by several mechanisms, including improved endothelial function, reduced arterial stiffness, and modulation of the autonomic nervous system to decrease sympathetic activity [11, 12]. These effects are complemented by significant weight loss, which itself contributes to reductions in systolic and diastolic blood pressures [13]. Additionally, GLP-1 RAs have been shown to attenuate oxidative stress and inflammatory pathways, further supporting vascular health [14, 15].

Clinical trials have demonstrated that GLP-1 RAs, such as liraglutide and semaglutide, reduce blood pressure across diverse populations with T2DM and obesity. However, variability in individual responses underscores the complexity of their effects and highlights the need to identify patient subgroups most likely to benefit from this therapy. Moreover, the durability and long-term implications of these blood pressure-lowering effects remain areas requiring further investigation. This review synthesizes peer-reviewed evidence available to date that elucidates the role of GLP-1 RAs in blood pressure regulation, and examines their mechanisms of action, clinical efficacy, and broader cardiovascular implications. By integrating findings from preclinical studies, randomized controlled trials, and meta-analyses, this review aims to provide a comprehensive understanding of the therapeutic potential of GLP-1 RAs in managing hypertension within the framework of diabetes and obesity care.

## 2. Mechanisms of GLP-1 Receptor Agonists in Blood Pressure Reduction

**Fig. 1.**
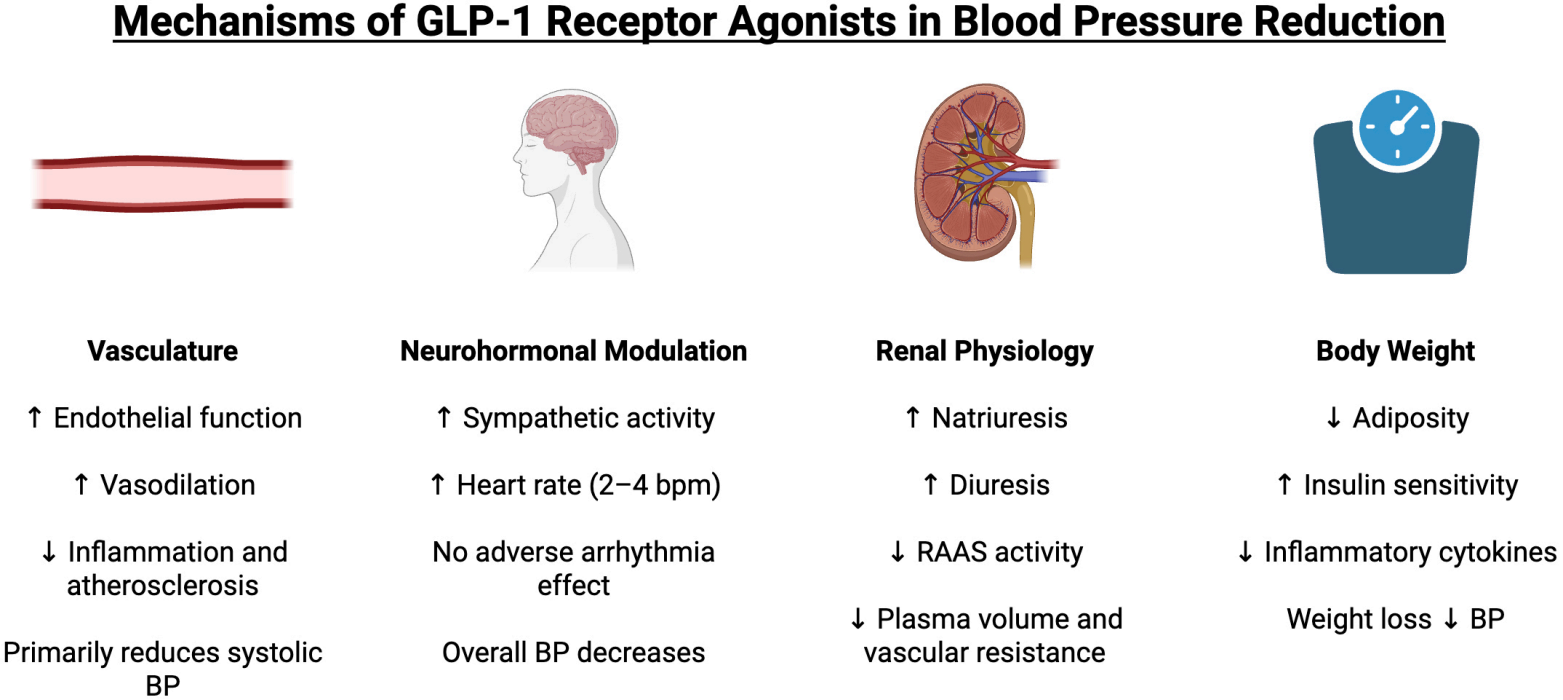
**Mechanisms of GLP-1 receptor agonists in blood pressure reduction**. GLP-1, glucagon-like peptide-1; BP, blood pressure; RAAS, renin-angiotensin-aldosterone system. Upward arrows (↑) denote an increase or enhancement, while downward arrows (↓) represent a decrease or suppression.

GLP-1 RAs decrease blood pressure through various direct and indirect mechanisms. These mechanisms include effects on the vasculature, neurohormonal modulation, renal physiology, and body weight, all of which contribute to reductions in systemic blood pressure (Fig. 1).

GLP-1 receptors are expressed at low levels in the heart and vasculature; still, activation of these receptors can induce beneficial vascular changes [16]. GLP-1 RAs have been shown to improve endothelial function and promote vasodilation, partly via nitric oxide (NO) dependent pathways. For instance, in studies of patients with diabetes, GLP-1 infusion or GLP-1 RA therapy enhanced endothelium-dependent vasodilation and increased NO release [17]. Basu *et al.* [18] observed that GLP-1 infusion improved acetylcholine-mediated vasorelaxation and even lowered diastolic blood pressure (BP) in type 1 diabetic patients. Additionally, GLP-1 RAs also exhibit anti-inflammatory and anti-atherosclerotic effects on the vasculature, which may further improve endothelial function [12, 19]. Notably, GLP-1 RAs tend not to significantly affect diastolic pressure in most patients, likely because their vasodilatory effect primarily impacts systolic pressure and large artery compliance.

GLP-1 RAs can also influence the autonomic nervous system, although the net impact on BP is complex. GLP-1 receptors in the brain may stimulate sympathetic outflow. Acute administration of native GLP-1 or GLP-1 RAs has been associated with increased heart rate, suggesting some degree of sympathetic activation [20, 21]. Preclinical studies indicate that GLP-1 in the paraventricular nucleus can raise blood pressure via sympathetic nervous activation. In clinical trials, GLP-1 RAs consistently produce a modest increase in heart rate of about 2–4 beats per minute [22]. This mild tachycardic effect is thought to be mediated by GLP-1 receptor activation in autonomic centers of the heart, and it occurs irrespective of the presence of hypertension. While increased heart rate is generally a counter-regulatory response that could blunt BP reduction, the overall blood pressure effect of GLP-1 RAs remains a net decrease, as other mechanisms such as vasodilation, natriuresis, and weight loss dominate. Some have raised concerns that chronic slight tachycardia might offset cardiovascular benefits, but the major outcome trials did not find a harmful impact of the heart rate increase. In summary, GLP-1 agonists likely engage sympathetic pathways, resulting in a small rise in heart rate but no adverse arrhythmic effect, while still allowing an overall blood pressure reduction through other means.

Another important mechanism by which GLP-1 RAs may lower blood pressure is through renal sodium and water excretion. GLP-1 receptors in the kidneys, specifically in the proximal tubule, can promote natriuresis and diuresis when activated. Studies have demonstrated that GLP-1 RA administration increases sodium excretion. For instance, Skov *et al*. [23] found that intravenous GLP-1 infusion in healthy men induced significant natriuresis and diuresis, accompanied by a reduction in plasma angiotensin II levels. This suggests GLP-1 signaling can downregulate the renin-angiotensin-aldosterone system (RAAS). Clinically, GLP-1 RAs have demonstrated direct natriuretic effects. A study in patients with type 2 diabetes showed that liraglutide acutely increased urinary sodium excretion despite no change in atrial natriuretic peptide levels, confirming a natriuretic action in humans [24]. By promoting sodium loss and possibly antagonizing RAAS, GLP-1 RAs reduce plasma volume and vascular resistance, contributing to lower blood pressure. Overall, the evidence strongly suggests GLP-1 receptor activation helps control blood pressure at least partly by improving renal sodium handling and reducing RAAS activity [12].

GLP-1 RAs are potent weight-reducing agents, and weight loss is a well-known strategy to lower blood pressure in overweight individuals. Reductions in adiposity lead to improved insulin sensitivity, decreased circulating insulin (hyperinsulinemia can raise BP by renal sodium retention), reduced inflammatory cytokines, and improved vascular function; all of which can lower blood pressure. On average, GLP-1 RA therapy produces significant weight loss over months; this likely mediates a portion of the blood pressure reduction. In fact, meta-regression analyses have found that the extent of BP lowering with GLP-1 RAs correlates with the magnitude of weight loss. A recent meta-analysis by Rivera *et al*. [25] noted that patients on GLP-1 RAs experienced modest systolic BP reductions in parallel with reductions in body mass index (BMI) and hemoglobin A1c, suggesting the BP effect is secondary to improvements in weight and metabolic control. Weight loss of 5–10% is known to significantly improve BP in hypertensive patients, as evidenced by lifestyle intervention studies [26]. Therefore, the substantial weight reduction induced by GLP-1 RAs is a key indirect mechanism for lowering BP. Importantly, GLP-1 RAs reduce blood pressure even before dramatic weight loss occurs, indicating other direct effects are also at play early in therapy [27]. But over the longer term, decreased adiposity, improved insulin sensitivity, and lower sympathetic drive from weight loss all contribute to sustained blood pressure improvements.

## 3. GLP-1 Agonists and Cardiovascular Outcomes

Large clinical trials have provided critical insights into the cardiovascular benefits of GLP-1 RAs, particularly their impact on blood pressure regulation. The results of these studies are summarized in Table 1 (Ref. [28, 29, 30, 31]).

**Table 1.** **Summary of randomized controlled trials evaluating the cardiovascular and blood pressure effects of GLP-1 receptor agonists**.

Study	Population	Intervention	Key findings
LEADER trial [28]	9340 T2DM patients with high cardiovascular risk	Liraglutide (1.8 mg/day) vs. placebo	Reduced MACE by 13% (HR: 0.87, 95% CI: 0.78–0.97, *p* = 0.01). SBP reduction: 1.2 mmHg (95% CI: 0.5–1.9). DBP reduction: 0.6 mmHg (95% CI: 0.2–1.0).
REWIND trial [29]	9901 T2DM patients (69% with prior cardiovascular disease)	Dulaglutide (1.5 mg/week) vs. placebo	MACE reduced by 12% (HR: 0.88, 95% CI: 0.79–0.99, *p* = 0.026). SBP reduction: 1.70 (95% CI: 1.33–2.07).
SUSTAIN-6 trial [30]	3297 T2DM patients with CV risk factors	Semaglutide (0.5 mg or 1 mg weekly) vs. placebo	MACE risk reduced by 26% (HR: 0.74, 95% CI: 0.58–0.95). SBP reduction: 2.6 mmHg (*p * < 0.001) in the 1 mg weekly group.
SURMOUNT-1 trial [31]	2539 adults with obesity (BMI ≥30) or overweight (BMI ≥27) with ≥1 weight-related condition, excluding diabetes	Tirzepatide (5 mg, 10 mg, or 15 mg weekly) vs. placebo	SBP reduction: 6.2 (95% CI: 4.8–7.7). DBP reduction: 4.0 (95% CI: 3.1–4.9).

HR, hazard ratio; SBP, systolic blood pressure; DBP, diastolic blood pressure; CI, confidence interval; CV, cardiovascular; MACE, major adverse cardiovascular events; T2DM, type 2 diabetes mellitus; BMI, body mass index.

### 3.1 The LEADER Trial

The LEADER trial was a double-blind, randomized, placebo-controlled study designed to evaluate the cardiovascular safety of liraglutide in 9340 patients with T2DM and high cardiovascular risk. Participants were required to have an HbA1c level of ≥7.0%, be at least 50 years old with cardiovascular disease, or be at least 60 years old with multiple cardiovascular risk factors. Exclusion criteria included individuals with recent acute coronary syndromes, New York Heart Association (NYHA) class IV heart failure, or a life expectancy of less than one year. Patients were randomized to receive either liraglutide (1.8 mg daily) or placebo in addition to standard care. The median duration of follow-up was 3.8 years. Results showed that liraglutide significantly reduced the primary composite outcome of major adverse cardiovascular events (MACE) by 13% compared to placebo (hazard ratio [HR] 0.87; 95% confidence interval [CI]: 0.78–0.97; *p* = 0.01). A secondary outcome revealed a mean systolic blood pressure reduction of 1.2 mmHg (baseline-adjusted *p* < 0.05). These reductions were associated with weight loss and improved vascular compliance. The study concluded that liraglutide provides cardiovascular protection while modestly reducing blood pressure, which may enhance its benefits in T2DM patients at high cardiovascular risk [28]. While the trial established liraglutide’s cardioprotective effect; limitations include a high risk population limiting generalization to lower-risk patients. Secondly, the absolute BP reduction was small relative to the magnitude of cardiovascular benefit suggesting multifactorial protective mechanisms beyond just BP lowering.

### 3.2 The REWIND Trial

The REWIND trial was a double-blind, randomized, placebo-controlled study conducted to evaluate the effects of dulaglutide on cardiovascular outcomes in 9901 patients with T2DM. The patient population included a broader spectrum of cardiovascular risk compared to LEADER, with 46% having no prior cardiovascular events. Participants were aged ≥50 years, with HbA1c levels of ≤9.5%, and were either on stable glucose-lowering therapy or newly diagnosed. Exclusion criteria included recent cardiovascular events and severe renal impairment (estimated glomerular filtration rate <15 mL/min/1.73 m^2^). Patients were randomized to receive dulaglutide (1.5 mg weekly) or placebo, with a median follow-up of 5.4 years. Dulaglutide significantly reduced the primary composite outcome of MACE by 12% (HR 0.88; 95% CI: 0.79–0.99; *p* = 0.026). A mean systolic blood pressure reduction of 1.7 mmHg (baseline-adjusted *p* < 0.01) was noted as a secondary outcome. These findings highlight the role of dulaglutide in improving cardiovascular outcomes and lowering blood pressure in T2DM patients with varying degrees of cardiovascular risk [29]. Interestingly, while overall MACE reduction was statistically significant; the benefit appeared most consistent for nonfatal stroke with less robust effects on myocardial infarction (MI) or cardiovascular (CV) death.

### 3.3 The SUSTAIN-6 Trial

The SUSTAIN-6 trial was a multicenter, double-blind, randomized, placebo-controlled study assessing the cardiovascular safety of Semaglutide in 3297 patients with T2DM and high cardiovascular risk. Inclusion criteria included patients aged ≥50 years with cardiovascular disease or ≥60 years with multiple cardiovascular risk factors. Exclusion criteria included NYHA class IV heart failure and recent major cardiovascular events. Patients were randomized to receive Semaglutide (0.5 or 1.0 mg weekly) or placebo over a median follow-up of 104 weeks. Semaglutide significantly reduced the primary outcome of MACE by 26% (HR 0.74; 95% CI: 0.58–0.95; *p* = 0.02). Additionally, systolic blood pressure decreased by an average of 2.6 mmHg (*p* < 0.001). These effects were likely mediated by weight loss (average 4.2 kg reduction, *p* < 0.001) and enhanced endothelial function. The study concluded that semaglutide provides both glycemic and cardiovascular benefits, with reductions in blood pressure as a secondary outcome contributing to its overall therapeutic profile [30]. Importantly, the trial was designed for non-inferiority rather than superiority and its relatively small sample size and short duration limit conclusions about long-term efficacy and safety. The modest BP reduction is difficult to disentangle from concurrent weight and glycemic effects.

### 3.4 SURMOUNT‑1 Trial

SURMOUNT-1 trial was a randomized, double-blind, placebo-controlled phase 3 trial evaluating the safety and efficacy of tirzepatide in 2539 obese (BMI ≥30) or overweight (BMI ≥27) adult patients with at least one weight-related comorbidity, excluding diabetes. Participants were randomly assigned to weekly subcutaneous tirzepatide 5 mg, 10 mg, or 15 mg, or placebo for 72 weeks with a 20-week dose-escalation period. The trial revealed substantial, dose-response reductions in body weight with 15.0%, 19.5%, and 20.9% weight reduction with 5 mg, 10 mg, and 15 mg, respectively versus 3.1% with placebo (*p* < 0.001). Tirzepatide reduced systolic blood pressure by up to 6.8 mmHg and diastolic pressure by up to 4.2 mmHg. This trial concluded that tirzepatide induces substantial weight loss and improves blood pressure highlighting its therapeutic potential for the management of obesity and cardiovascular risk [31]. While impressive, the trial’s primary endpoint was weight loss not cardiovascular outcomes and BP effects were secondary. Exclusion of patients with diabetes reduces generalizability to broader cardiometabolic populations and trial conduct with frequent visits and titration support may overestimate adherence and efficacy compared with real-world practice.

## 4. GLP-1 Agonists vs. Traditional Antihypertensive Therapies

Given the modest blood pressure reductions with GLP-1 RAs, a natural question is how they compare to conventional antihypertensive drugs. In general, the BP-lowering effect of GLP-1 RAs is less than that of standard antihypertensive medications when used in monotherapy. Most first-line antihypertensive drug classes such as ACE inhibitors, angiotensin receptor blockers, thiazide diuretics, and calcium channel blockers lower systolic BP by roughly 5–15 mmHg on average in hypertensive patients, depending on baseline BP and dose [32, 33, 34, 35]. Even newer agents like SGLT2 inhibitors typically lower SBP ~4–5 mmHg [36, 37, 38, 39]. Thus, GLP-1 RAs should not be viewed as replacements for dedicated BP medications when significant BP lowering is required. However, GLP-1 RAs can provide an additive BP-lowering effect when used alongside conventional antihypertensives.

When considering efficacy, GLP-1 RAs alone are generally insufficient for treating moderate or severe hypertension, but they can meaningfully improve BP in patients whose levels are just above goal despite standard therapy. An interesting comparison is with weight-loss medications, in a network meta-analysis of weight management trials in overweight hypertensives, GLP-1-based agents were associated with the largest BP reductions among pharmacotherapies [40]. Tirzepatide led the pack with ~6.5 mmHg SBP and ~3.6 mmHg DBP lowering vs placebo, outperforming older weight-loss drugs like orlistat or phentermine-topiramate which lowered SBP by ~2–4 mmHg [40]. This underscores that for an obese hypertensive patient, a GLP-1 RA can provide an antihypertensive benefit comparable to adding a mild BP drug, especially as part of a weight-centric strategy.

Beyond efficacy, the tolerability and suitability of GLP-1 RAs differ substantially from traditional antihypertensive agents. GLP-1 RAs are associated with gastrointestinal side effects such as nausea, vomiting and diarrhea most prominent during initiation and dose titration. These adverse events along with their higher cost can limit long-term adherence and make them less practical for patients who do not also have diabetes or obesity. By contrast, conventional antihypertensives have long-established safety profiles in diverse populations with hypertension. However, they do not confer metabolic or weight-reducing benefits. This highlights that patient selection is crucial. GLP-1 RAs are especially valuable for obese or diabetic hypertensive patients.

The most significant advantage of utilizing GLP-1 RAs is their metabolic benefits along with the modest BP reductions. The significant weight loss, improved glycemic control, favorable impact on lipid profile, reduction in inflammation and improved endothelial function make it a wonderful choice of lowering cardiovascular risk. No traditional anti-hypertensive offers this wide range of benefits. Therefore, as an adjunct, GLP-1 RAs can be particularly beneficial in patients with both hypertension and diabetes and/or obesity.

There are, of course, no indications or recommendations for GLP-1 RA use solely for hypertension control as important limitations to using them for blood pressure management exist. First, they are less potent at lowering BP than dedicated antihypertensive drugs, as noted. A patient with significantly elevated BP will still require standard antihypertensive therapy; a GLP-1 RA would be insufficient as monotherapy to reach BP targets in most cases. Second, GLP-1 RAs are costly, which makes them impractical to use solely for mild BP elevation if the patient has no other indication. Third, side effects of GLP-1 RAs need to be considered with the most common ones being gastrointestinal (nausea, vomiting, diarrhea), especially during initiation and titration.

Current guidelines underscore this adjunctive role. The American Diabetes Association recommends GLP-1 RAs with proven cardiovascular benefit such as liraglutide, semaglutide or dulaglutide for patients with type 2 diabetes and established atherosclerotic cardiovascular disease or high cardiovascular risk irrespective of HbA1c level or background therapy. Similarly, the 2023 European Society of Hypertension guidelines acknowledge the favorable metabolic and weight-loss effects of GLP-1 RAs but do not endorse them as first-line antihypertensive drugs. These recommendations reinforce the concept that GLP-1 RAs should complement not replace the conventional antihypertensives when cardiovascular risk reduction is the therapeutic priority.

## 5. Conclusions

In summary, GLP-1 RAs complement but do not replace traditional antihypertensive therapy. Their modest BP reduction can be thought of as equivalent to adding a mild antihypertensive. The real value of GLP-1 RAs lies in their multi-factorial benefits. For the growing population of patients with the co-morbid conditions of hypertension, diabetes, and obesity, GLP-1 RAs represent an opportunity to simultaneously treat multiple risk factors with one agent.
